# Elucidating the Glucokinase Activating Potentials of Naturally Occurring Prenylated Flavonoids: An Explicit Computational Approach

**DOI:** 10.3390/molecules26237211

**Published:** 2021-11-28

**Authors:** Kolade Olatubosun Faloye, Boris Davy Bekono, Emmanuel Gabriel Fakola, Marcus Durojaye Ayoola, Oyenike Idayat Bello, Oluwabukunmi Grace Olajubutu, Onikepe Deborah Owoseeni, Shafi Mahmud, Mohammed Alqarni, Ahmed Abdullah Al Awadh, Mohammed Merae Alshahrani, Ahmad J. Obaidullah

**Affiliations:** 1 Department of Chemistry, Faculty of Science, Obafemi Awolowo University, Ile-Ife 220282, Nigeria; emmseg98@yahoo.com; 2 Department of Physics, Ecole Normale Supérieure, University of Yaoundé 1, Yaoundé BP 812, Cameroon; borisbekono@gmail.com; 3 Institute of Pharmaceutical Sciences, Albert-Ludwigs-Universität, 79104 Freiburg, Germany; 4 Department of Pharmacognosy, Faculty of Pharmacy, Obafemi Awolowo University, Ile-Ife 220282, Nigeria; ayosther@yahoo.com (M.D.A.); oyenikebello@gmail.com (O.I.B.); owoseenionikepe@gmail.com (O.D.O.); 5 Department of Pharmaceutics, Faculty of Pharmacy, Obafemi Awolowo University, Ile-Ife 220282, Nigeria; olajubutuoluwabukunmi@gmail.com; 6 Genetic Engineering and Biotechnology, University of Rajshahi, Rajshahi 6205, Bangladesh; shafimahmudfz@gmail.com; 7 Department of Pharmaceutical Chemistry, College of Pharmacy, Taif University, P.O. Box 11099, Taif 21944, Saudi Arabia; m.aalqarni@tu.edu.sa; 8 Department of Clinical Laboratory Sciences, Faculty of Applied Medical Sciences, Najran University, P.O. Box 1988, Najran 61441, Saudi Arabia; a-21-ksa@hotmail.com (A.A.A.A.); mmalshahrani@nu.edu.sa (M.M.A.); 9 Drug Exploration and Development Chair (DEDC), Department of Pharmaceutical Chemistry, College of Pharmacy, King Saud University, Riyadh 11451, Saudi Arabia; aobaidullah@ksu.edu.sa; 10 Department of Pharmaceutical Chemistry, College of Pharmacy, King Saud University, Riyadh 11451, Saudi Arabia

**Keywords:** glucokinase activators, prenylated flavonoids, molecular docking, molecular dynamics simulation, density functional theory, ADMET

## Abstract

Glucokinase activators are considered as new therapeutic arsenals that bind to the allosteric activator sites of glucokinase enzymes, thereby maximizing its catalytic rate and increasing its affinity to glucose. This study was designed to identify potent glucokinase activators from prenylated flavonoids isolated from medicinal plants using molecular docking, molecular dynamics simulation, density functional theory, and ADMET analysis. Virtual screening was carried out on glucokinase enzymes using 221 naturally occurring prenylated flavonoids, followed by molecular dynamics simulation (100 ns), density functional theory (B3LYP model), and ADMET (admeSar 2 online server) studies. The result obtained from the virtual screening with the glucokinase revealed arcommunol B (−10.1 kcal/mol), kuwanon S (−9.6 kcal/mol), manuifolin H (−9.5 kcal/mol), and kuwanon F (−9.4 kcal/mol) as the top-ranked molecules. Additionally, the molecular dynamics simulation and MM/GBSA calculations showed that the hit molecules were stable at the active site of the glucokinase enzyme. Furthermore, the DFT and ADMET studies revealed the hit molecules as potential glucokinase activators and drug-like candidates. Our findings suggested further evaluation of the top-ranked prenylated flavonoids for their in vitro and in vivo glucokinase activating potentials.

## 1. Introduction

Glucokinase is a hexokinase enzyme involved in glucose phosphorylation during carbohydrate metabolism. The enzyme acts as a glucose sensor for regulating insulin secretion by the pancreas and as a marker for the network of glucose-sensitive neural and endocrine cells involved in glucose homeostasis [1]. Pancreatic glucokinase results in glucose-stimulated insulin secretion (GSIS) by detecting the variation in glucose levels and stimulating the release of insulin. An increase in glucose concentration results in increased cellular adenosine triphosphate (ATP), which is implicated in the closure of ATP-sensitive potassium (KATP) channels, depolarization of the β-cell, and calcium (Ca^2+^) influx through voltage-gated Ca^2+^ channels thereby stimulating the release of insulin [2,3]. In the liver, glucokinase is involved in promoting glucose uptake and its conversion to glycogen for energy storage [4,5].

Abnormal functionalities of glucokinase enzymes result from the delineation of its binding sites due to genetic mutation. This results in a gradual decline in pancreatic β-cell mass, which causes defective insulin secretion and increased hepatic glucose output, thereby leading to severe pathological conditions such as maturity-onset diabetes of the young (MODY), persistent hyperinsulinemia hypoglycaemia of infancy (PHHI-GK), and permanent neonatal diabetes mellitus (PNDM) [6]. Therefore, the use of glucokinase activators can result in blood glucose level reduction in type 2 diabetes mellitus patients [7].

Glucokinase activators are bioactive molecules that bind to the enzyme’s allosteric site, thereby improving glycemic control and glucose homeostasis through the stimulation of insulin secretion and glycogen synthesis [8]. They further control the super-open and closed conformational transitions of the glucokinase enzyme, which results in the promotion of its activity in the uptake of excess blood glucose [9]. Recently, glucokinase activators have been identified as new therapeutic agents for the management of type 2 diabetes. However, the vast majority of potential activators developed have been found to show side effects during clinical trials [10]. These side effects include hypoglycaemia, dyslipidaemia, hypertriglyceridemia, fatty liver induction, hepatic steatosis, and different microvascular complications [11,12]. Hence, there is a need to investigate medicinal plants for their bioactive constituents that could serve as potent glucokinase activators with lesser or no side effects in the management of diabetes.

Over time, plants have been identified as a source of different classes of phytochemicals that possesses excellent bioactivity and are essential for drug development and other therapeutic purposes. Prenylated flavonoid is a sub-class of flavonoid that is, in nature, low in abundance, safe, and non-poisonous in medicinal plants [13]. They have one or more isoprene unit that binds to the different positions of the aromatic ring of flavonoids, thereby enhancing its bioactivities against a wide range of diseases [14,15,16]. Prenylated flavonoids like xanthohumol, sophoraflavanone D, kushenol C, morusinol, and artonins A and B have been reported to possess anticancer, antibacterial, antidiabetic, anti-inflammatory, and antitrypanosomal activities [17,18]. Prenylated flavonoids, with their proven various important biological activities, are therefore, being considered to be potential glucokinase activators.

Drug discovery using computational methods like molecular docking, molecular dynamic simulation, and density functional theory are used in the discovery of large classes of compounds with various biological activities and have been found to be economical and effective [19,20,21]. Molecular docking and molecular dynamics simulation help in predicting the binding affinity and the stability of ligands in the binding pocket of target receptors [22]. Density functional theory is a computational method that is useful in predicting the pharmacological potentials and electronic properties of hit molecules in the treatment of diseases [23].

Therefore, this study was designed to discover potential glucokinase activators from naturally occurring prenylated flavonoids through the use of molecular docking methods. Binding affinity, binding modes, stability, electronic, and drug-likeness properties of the drug candidates will be elucidated through molecular dynamic simulation, density functional theory calculations, and ADMET methods.

## 2. Results and Discussion

### 2.1. Validation of Docking Protocol

**Figure 1:**
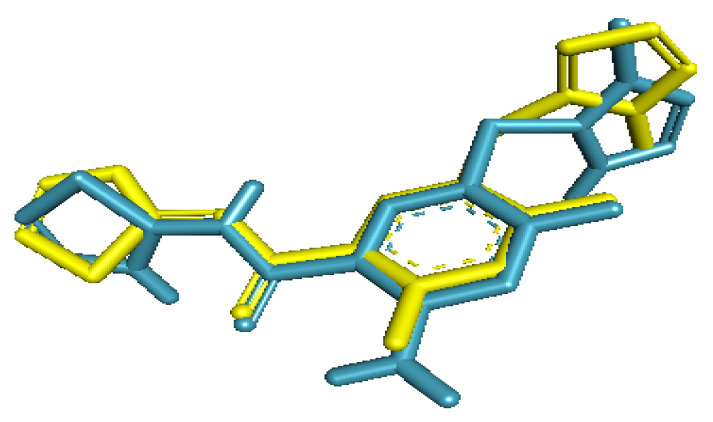
Conformation of native ligand (yellow) and re-docked ligand (blue) showing similar poses in the active site of glucokinase enzyme.

The re-docked ligand formed a similar conformational pose when compared with the native ligand in the glucokinase enzyme. The re-docked ligand was almost superimposed on the inbound ligand. Furthermore, the RMSD obtained between the native ligand and the re-docked ligand was 1.58 Å (Figure 1).

Additionally, all the amino acids residues (Tyr61, Val62, Arg63, Ser64, Thr65, Pro66, Gln98, Ile159, Met210, Ile211, Tyr214, Tyr215, His218, Cys220, Met235, Arg250, Leu451, Val452, Val455, and Ala456) that interacted with the co-crystallized ligand correctly matched those obtained between the re-docked ligand and the enzyme when viewed with PyMol [24].

### 2.2. Molecular Docking Analysis of Prenylated Flavonoids with Glucokinase Enzyme

Molecular docking studies was an efficient method used in predicting pharmacological potentials of naturally occurring chemical compounds against life-threatening diseases. In our study, we performed molecular docking studies of 221 prenylated flavonoids and the selection cut-off point for promising glucokinase activators was set to −9.0 kcal/mol. The resulting 11 ligands were further reduced based on their physicochemical properties and pan-assay interference compounds (PAINS) filter test to predict their possible drug candidacy. Finally, four top-ranked molecules were selected based on their high binding energies ranging from −9.4 kcal/mol to −10.1 kcal/mol (Table 1).

**Figure 2:**
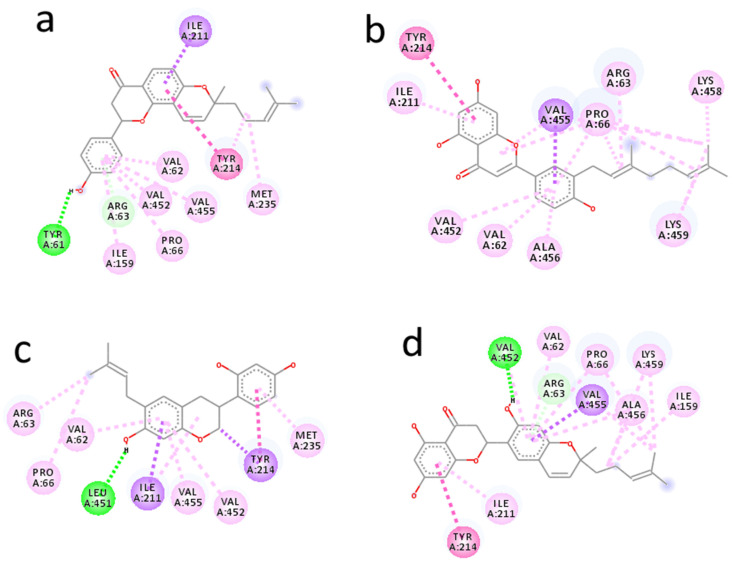
Interaction diagram of arcommunol B (**a**), kuwanon S (**b**), manuifolin H (**c**), and kuwanon S (**d**) with glucokinase enzyme.

Arcommunol B isolated from *Artocarpus communis* elicited the best binding energy of −10.1 kcal/mol. The hydrogen atom on the benzene ring of arcommunol B established a hydrogen bond interaction with Tyr61 at 2.19 Å. The ligand was further stabilized at the active site of the enzyme by forming ten hydrophobic interactions with nine amino acid residues (Val62, Arg63, Pro66, Ile159, Ile211, Tyr214, Val452, and Val455). Additionally, the arcommunol B moiety established pi-interactions (pi-sigma and pi-alkyl) with Val62, Arg63, Pro66, Ile159, Ile211, Tyr214, Met235, Val452, and Val455 (Figure 2a).

Kuwanon S identified from *Morus alba* elicited binding energy of −9.6 kcal/mol. The kuwanon F moiety was stabilized by participating in seventeen hydrophobic interactions with ten amino acid residues (Val62, Arg63, Pro66, Ile211, Tyr214, Val452, Val455, Ala456, Lys458, and Lys459). It further established pi-interactions with Val62, Arg63, Pro66, Ile211, Tyr214, Val452, Val455, Ala456, Lys458, and Lys459. However, no hydrogen bond interaction was formed between kuwanon S and the amino acid residues at the active site of the receptor (Figure 2b).

Manuifolin H obtained from *Maackia tenuifolia* had binding energy of −9.5 kcal/mol. One hydrogen bonding interaction was formed between the hydrogen atom on the benzene ring of manuifolin H and Leu451 at 2.05 Å. The ligand was further stabilized at the receptor’s active site by forming ten hydrophobic interactions with eight amino acid residues (Val62, Arg63, Pro66, Ile211, Tyr214, Met235, Val452, and Val455), while pi-interactions were established between manuifolin H and Val62, Arg63, Pro66, Ile211, Tyr214, Met235, Val452, and Val455 (Figure 2c).

Kuwanon F from *Morus alba* had a considerably high binding energy of −9.4 kcal/mol. The hydrogen atom on the aromatic ring of kuwanon F moiety formed a hydrogen bond interaction with Val452 at 2.82 Å. Furthermore, the ligand was stabilized by forming hydrophobic interactions with Val62, Arg63, Pro66, Ile159, Ile211, Tyr214, Val452, and Val455, while pi-sigma and pi-alkyl interactions were established with Val62, Arg63, Pro66, Ile159, Ile211, Tyr214, Val455, Ala456, and Lys459 (Figure 2d).

The results showed that the hit molecules selected had common hydrophobic interactions with Val62, Arg63, Pro66, Ile211, Tyr214, and Val452. Additionally, they established similar pi-interactions with Val62, Arg63, Pro66, Ile211, Tyr214, Val452, and Val455. The hydrogen bonding, hydrophobic, and pi-interactions formed between the ligands were responsible for the good binding energy and unique stability established by the ligands at the glucokinase enzyme’s binding pocket.

A lesser hydrogen bonding interaction and higher hydrophobic interaction signified that the latter have a greater contribution to the binding affinity and stability of a ligand in an enzyme’s binding pocket [25]. In this study, only one hydrogen bonding interaction was observed for arcommunol B, manuifolin H, and kuwanon F, while none was observed for kuwanon S. This indicated that hydrophobic interaction was more prevalent and contributed exceedingly to the binding affinity and stability of the ligands in the glucokinase enzyme’s binding pocket.

Furthermore, the existence of a pi-alkyl interaction showed the formation of a pi-electron cloud between the alkyl and aromatic group of a ligand and amino acid residues at the binding pocket of the target receptor [26]. It helped to consolidate on the stability of the ligand in the enzyme’s binding pocket. In our study, the stability of the ligands in the binding pocket of glucokinase was largely due to the existence of considerable higher numbers of pi-alkyl interactions formed in the docked protein-ligand complexes. Additionally, pi-sigma interactions facilitated the charge transfer and insertion of drugs in the binding site of an enzyme [27]. Hence, the unique stability established by top-ranked molecules in the binding pocket of the glucokinase enzyme could be attributed to the existence of pi-sigma interaction.

Generally, the binding energies of arcommunol B, kuwanon S, manuifolin H, and kuwanon F showed that they were potential antidiabetic constituents and may be responsible for the antidiabetic activity of *Artocarpus communis* and *Morus alba* [28,29]. Furthermore, the binding energy arcommunol B, kuwanon S and manuifolin H were higher when compared with those obtained for guggultetrol, catechin, and kaempferol in previous studies [30,31,32].

### 2.3. Molecular Dynamics Simulation

**Figure 3:**
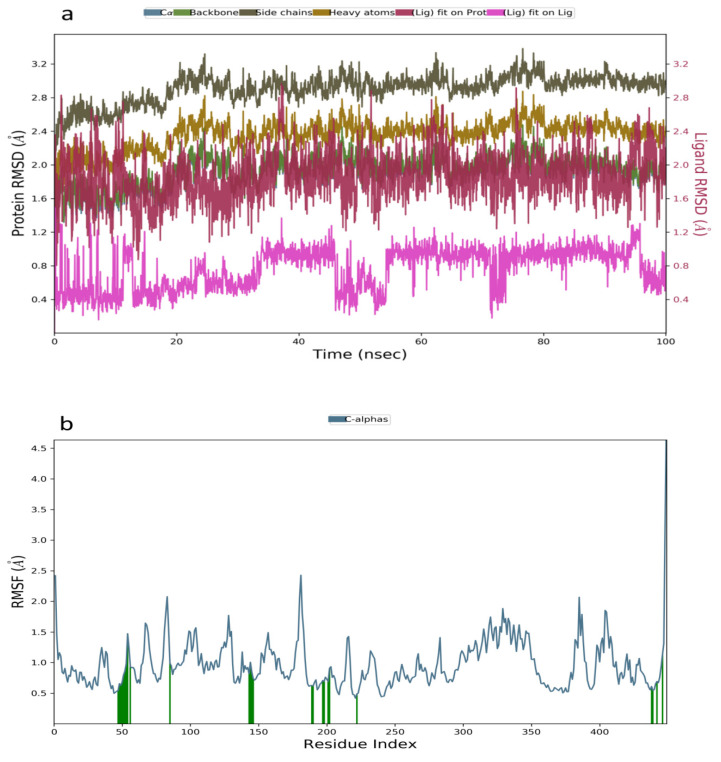

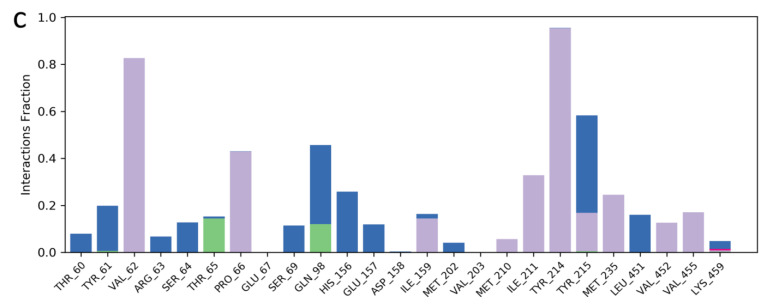
Molecular dynamics trajectory analysis of arcommunol B −1V4S: (**a**) RMSD of protein-ligand complex, (**b**) RMSF of protein-ligand complex, and (**c**) histogram of protein-ligand contacts.

Molecular dynamic simulation was a suitable method to validate the binding interaction and efficacy of ligands in drug discovery and development. In this study, the MDS of the best-docked poses of promising prenylflavonoids (arcommunol B, kuwanon F, kuwanon S, and manuifolin H) was carried out to elucidate the stability of the docked complexes in the glucokinase enzyme. The RMSD plot obtained from the MD simulation of arcommunol B−1V4S complex indicated that the simulated system was stable with the RMSD value that ranged from 0.4–1.2 Å (Figure 3a). Additionally, the stability in the simulated system was further confirmed through the RMSF value obtained from the RMSF plot that ranged between 0.5 to 2.5 Å (Figure 3b). Additionally, arcommunol B formed a hydrophobic interaction with Val62, Pro66, Ile211, Tyr214, Met235, and Val455, while a water bridge was formed with Tyr61, Gln98, His156, Tyr215, and Leu451 (Figure 3c).

**Figure 4:**
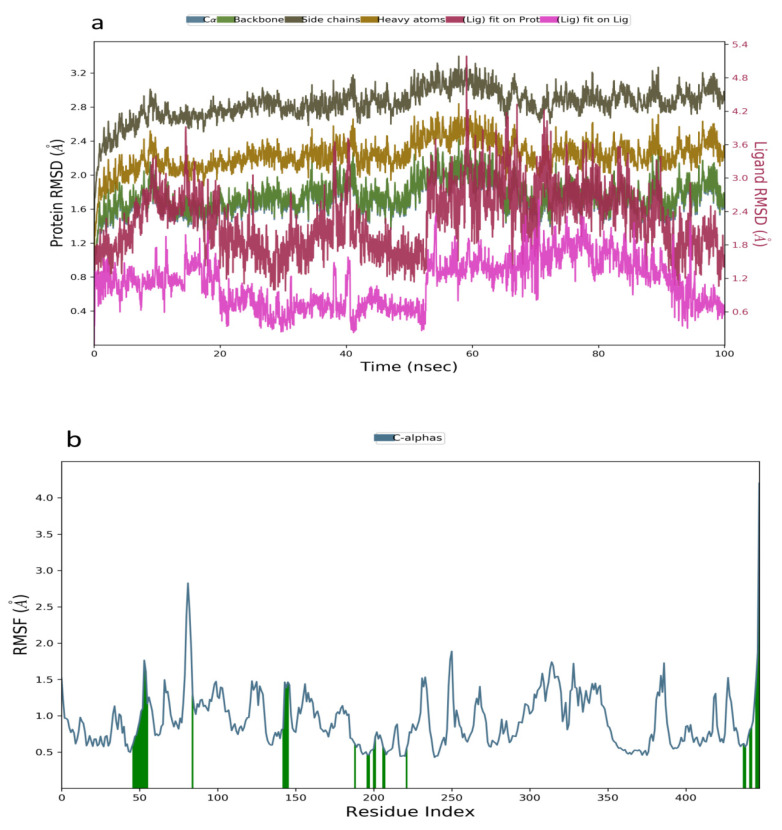

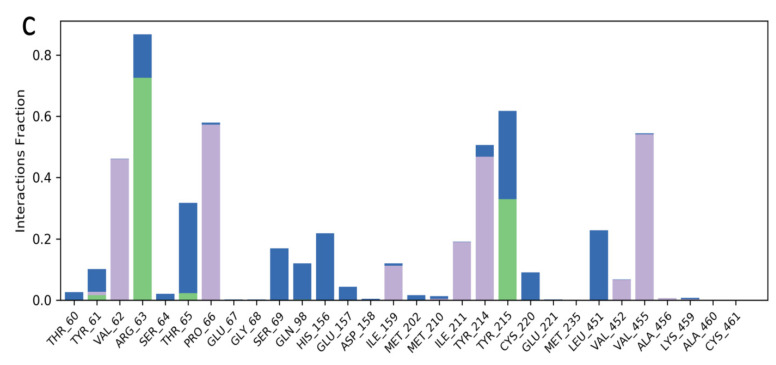
Molecular dynamics trajectory analysis of Kuwanon S−1V4S: (**a**) RMSD of protein-ligand complex, (**b**) RMSF of protein-ligand complex, and (**c**) histogram of protein-ligand contacts.

In the analysis of the MDS data acquired for kuwanon S−1V4S complex, the RMSD plot indicated a stable system from 0–20 ns, followed by a slight decrease to 0.6 Å and a stable system between 21–56 ns. A sharp pleateau and stable system was observed at 0.6–1.2 Å, within 57–100 ns (Figure 4a). The RMSF value obtained from the RMSF plot ranged from 0.5–2.9 Å, further establishing the stability of the simulated complex (Figure 4b). Additionally, the binding interactions analysis between kuwanon S and the residues at the active site of the glucokinase enzyme showed the formation of hydrophobic interaction with Val62, Pro66, Ile159, Ile211, Tyr214, and Val455, while a water bridge was formed with Arg63, Thr65, Ser69, Gln98, His156, Tyr215, Cys220, and Leu451. Furthermore, kuwanon S was stabilized throughout the entire simulation period by forming a hydrogen bond with Arg63 and Tyr215 (Figure 4c).

**Figure 5:**
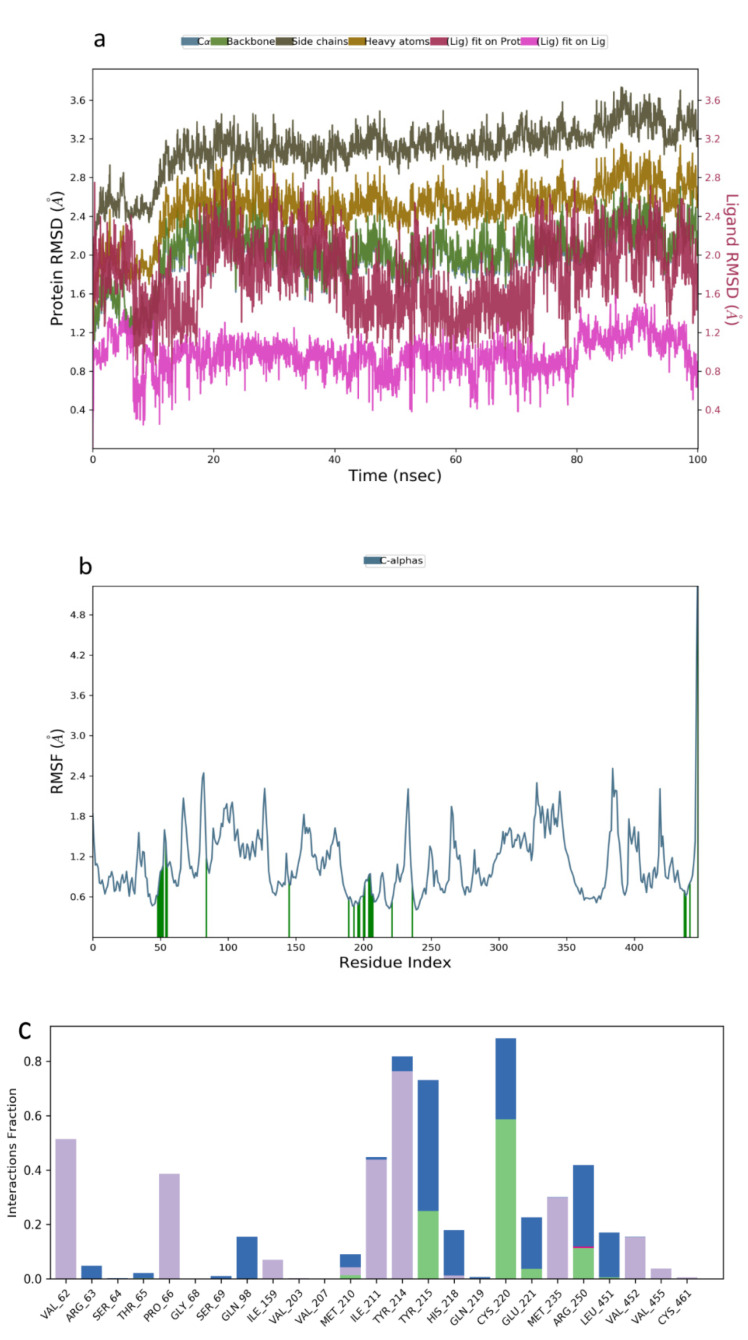
Molecular dynamics trajectory analysis of manuifolin H−1V4S: (**a**) RMSD of protein-ligand complex, (**b**) RMSF of protein-ligand complex, and (**c**) histogram of protein-ligand contacts.

Table 1. V4S showed an increase in the simulated system from 0–6 ns followed by stable system between 7–80 ns. A slight increase and stable system were observed between 8–100 ns (Figure 5a). Additionally, the RMSF plot obtained from 0.5–2.7 Å fur-ther confirmed the stability of the complex throughout the 100 ns simulation period (Figure 5b). The binding interactions obtained between manuifolin H and the amino acid residues at the binding site of the receptor indicated the stabilization of the ligand by participating in hydrogen bonding interactions with Tyr215, Cys220, and Arg250, while hydrophobic interactions were formed with Val62, Pro66, Ile211, Tyr214, Met235, and Val452. A water bridge was also formed between Gln98, Tyr215, His218, Cys220, Glu221, Arg250, and Leu451 (Figure 5c).

**Figure 6:**
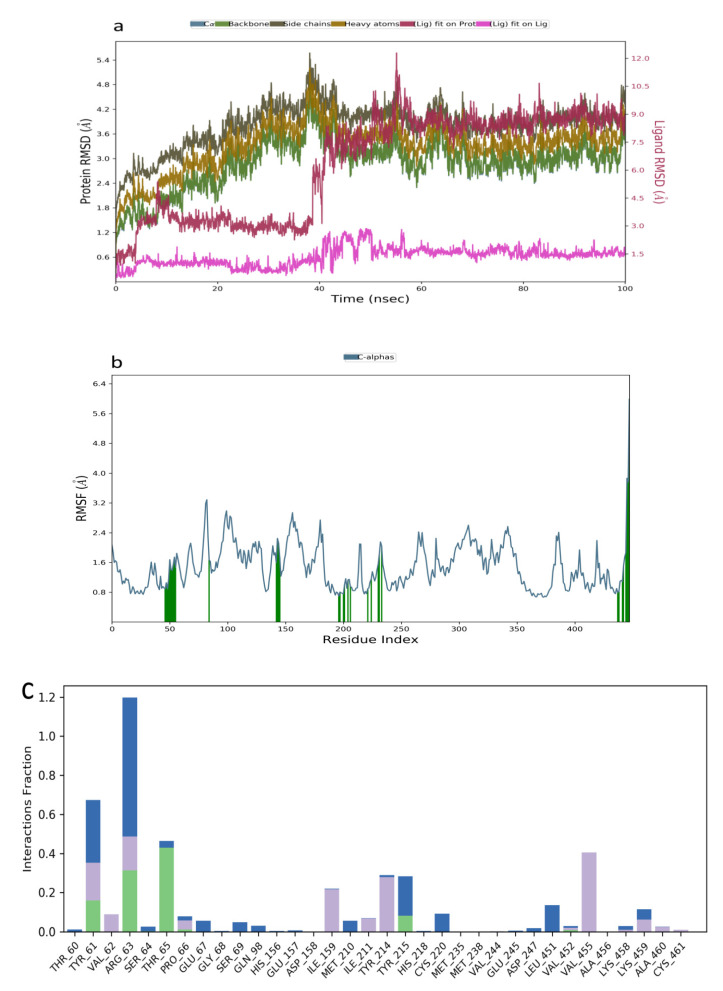
Molecular dynamics trajectory analysis of kuwanon F−1V4S: (**a**) RMSD of protein-ligand complex, (**b**) RMSF of protein-ligand complex, and (**c**) histogram of protein-ligand contacts.

The RMSD plot obtained for the kuwanon F−1V4S that indicated stability was attained between 0–40 ns, while a slight fluctuation was observed between 41–50 ns followed by a stable system from 51–100 ns (Figure 6a). The RMSF plot acquired ranged from 0.5–3.6 Å, indicating a moderate simulated system (Figure 6b). Additionally, the interaction obtained between kuwanon F and the residues at the active site of the receptor indicated the formation of a hydrophobic interaction with Tyr61, Arg63, Ile159, Tyr214, and Val455. The ligand was further stabilized by forming a hydrogen bonding interaction with Tyr61, Arg63, and Thr65. Furthermore, a water bridge was formed between the kuwanon F and Tyr61, Arg63, and Tyr215 (Figure 6c).

The molecular dynamics simulation of the top-ranked molecules with the active site of glucokinase supported the results obtained from the molecular docking studies. The formation of hydrophobic and hydrogen bonding interaction was known to contribute significantly to the stable state and binding energy of a ligand in the binding pocket of an enzyme [33]. Additionally, the formation of a water bridge played a significant role in the binding of the ligands to the amino acid residues at the receptor’s active site [33]. Our findings established that the ligands exhibited high binding poses in the binding pocket of the glucokinase enzyme. Furthermore, the stable backbone RMSD and the lesser fluctuation indicated the stability of the docked complexes throughout the 100 ns simulation period. However, arcommunol B was identified as the best hit molecule, considering the good binding energy that resulted from its high hydrophobic interaction and water bridge formation with amino acid residues at the active site of glucokinase. Generally, all the hit molecules showed promising glucokinase activating potentials based on their binding energy, interactions, and stability observed with the glucokinase enzyme throughout the entire molecular dynamics simulation period.

### 2.4. Analysis of Binding Free Energy

MM-GBSA is an efficient method to evaluate the binding affinity of hit molecules to receptors. In this study, the MM-GBSA binding energy (∆G_bind_) for each protein-ligand simulated system was calculated based on the MDS trajectories acquired throughout the 100 ns simulation period. arcommunol B elicited the highest average binding energy of −70.23 kcal/mol, followed by kuwanon S with −54.86 kcal/mol, followed by Manuifolin H with −37.34 kcal/mol, and Kuwanon F with −26.76 kcal/mol (Table 2). Other important energy components that contributed significantly to the total binding affinity of the ligand are given in Table 3.

### 2.5. Molecular Modeling and Quantum Chemical Calculations

The frontier molecular orbitals (HOMO and LUMO) of molecules are important in understanding their chemical reactivity and stability. These orbitals are known to play an active role during chemical reactions or interactions with other species [34]. The energy gap which results from these orbitals is a valuable parameter in predicting the chemical reactivity of such molecules [35]. The molecular electrostatic potential (MESP) is another valuable computational tool employed in the precise prediction and identification of electron-rich and electron deficient regions. It reveals sites that are preferred for electrostatic dominated non-covalent interactions and aids the understanding of electronic configuration of molecules [36,37].

The highest occupied molecular orbital (HOMO) describes the electron donating ability of the molecule, while the lowest unoccupied molecular orbital (LUMO) is associated with the electron accepting ability of the molecule. This implies that a higher E_HOMO_ value suggests a high donating ability, while a low E_LUMO_ value suggests better accepting ability [38]

The energy gap (ΔE), which is the difference between the E_LUMO_ and E_HOMO_, is a measure of the reactivity and stability of a molecule [39]. A higher energy gap is often associated with high stability and low reactivity, while a lower energy gap suggests lower stability and high reactivity [40].

**Figure 7:**
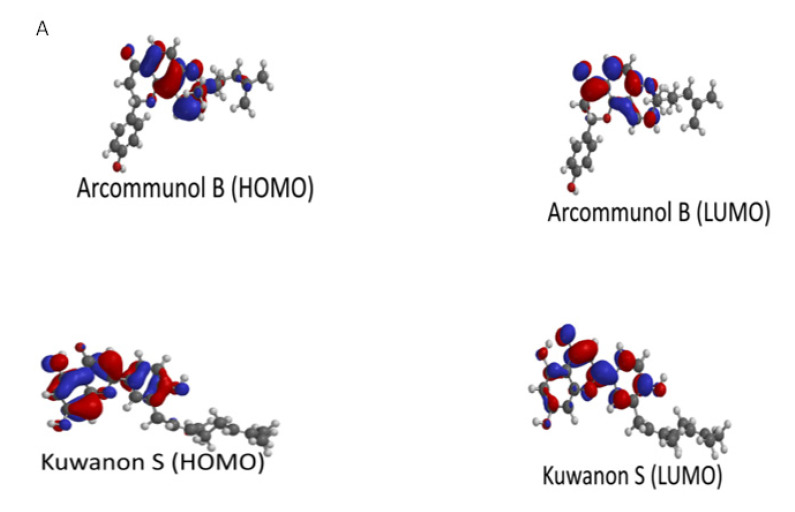

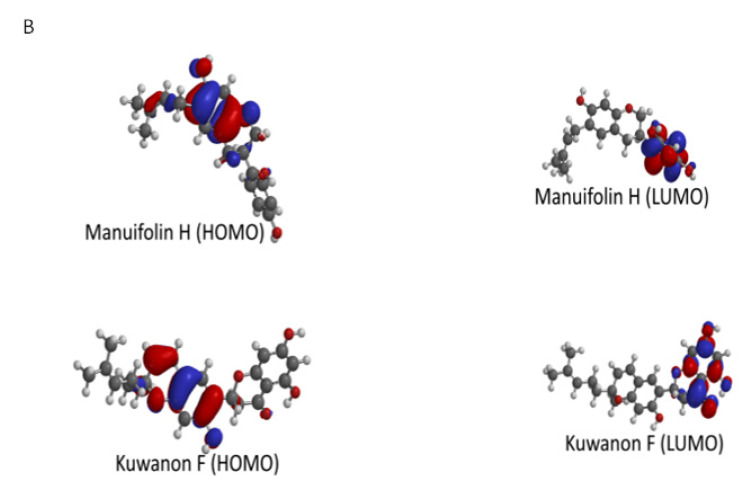
(**A**) Illustration of HOMO and LUMO of Arcommunol B and Kuwanon S. (**B**) Illustration of HOMO and LUMO of Manuifolin H and Kuwanon F.

Manuifolin H had the highest energy gap, while kuwanon F had the lowest energy gap (Table 3). This suggested that the former had the least reactivity and highest stability, while the latter was likely the most reactive compound, as well as the least stable. The HOMO and LUMO plots of kuwanon F, arcommunol B, kuwanon S, and manuifolin suggested that the molecules were delocalized over the aromatic ring system and π-network within the compounds (Figure 7A,B).

In an effort to fully understand the electronic properties of the compounds, the electronic parameters such as the chemical potential (μ), chemical hardness (η), and electrophilcity index (ω) were computed.

The chemical hardness of a molecule was related to its energy gap. It could be closely referred to as the opposition of a molecule to exchange electron density with the environment [41]. Hard molecules often possess a large energy gap while soft molecules often possess a small energy gap [42]. The energy gap and the hardness of the compounds followed the following order: manuifolin H > arcommunol B > kuwanon S > kuwanon F. Hence, manuifolin H was likely the hardest molecule, while kuwanon S was likely the softest (Table 3).

Chemical potential referred to the changes in the energy of a molecule, with respect to the electron number at a fixed potential. It was related to the probability of a molecule to exchange electron density with the environment at the ground state, and it was linked to the electrophilcity index of molecules [42]. Electrophilcity index measured the energy stabilization of a molecule when it acquired an extra amount of electronic density from the environment. Strong electrophiles had high chemical hardness and low chemical potential, while the converse was true for weak electrophiles. In effect, strong electrophiles possessed high electrophilcity index values, while weak electrophiles had lower electrophilcity values [41]. All the compounds under consideration had high electrophilicity index values (Table 4). Moreover, the values were higher than 0.8, hence they could be classified as strong electrophiles [41]. Kuwanon S was the strongest electrophile, closely followed by arcommunol B and kuwanon F, while manuifolin H had the lowest value. Therefore, it was likely the weakest electrophile among the compounds.

**Figure 8:**
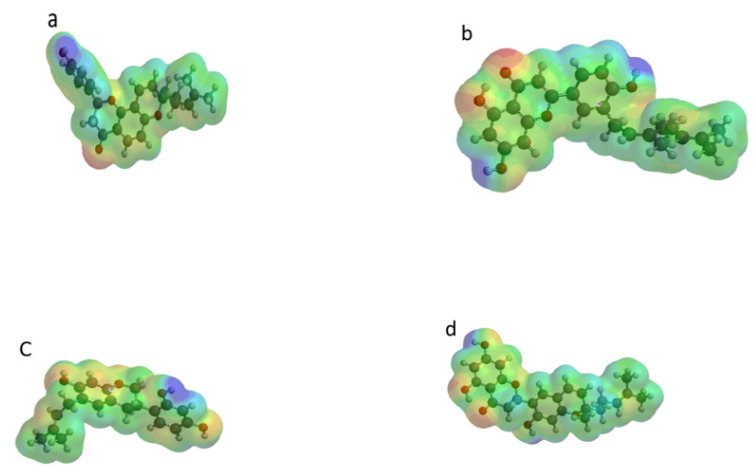
MESP of (**a**) Arcommunol B, (**b**) Kuwanon S, (**c**) Manuifolin H, and (**d**) and Kuwanon F.

Molecular electrostatic potential (MESP) was often used to investigate the chemical reactivity of molecules [42]. It was a plot of electrostatic potential over constant electron density of molecular systems. Its usefulness lied in the fact that it provided a visual representation of the molecular size, shape, and potential (positive, negative, and neutral) of molecules using a color grading scheme [42]. It was often used in identifying the reactive sites of the nucleophilic and electrophilic attack in bonding interactions, as well as in the field of biological recognition [41]. The MESP of the four compounds were represented with blue and red regions; with the former signifying the more electropositive regions of the compounds (regions that are electron poor) while the red regions signified the electronegative (i.e., electron rich regions, which were likely to be electrophilic centers) (Figure 8). The presence of these regions made the molecules be more similar to that of a hydrogen bond donor; this was relevant in the molecule’s biological activity as the ability of a drug to bind to a targeted receptor was largely influenced by the electrostatic potential due to the receptor being more likely to possess both sides. The electron-rich centres were often associated with negaive electrostatic potential, while the electron deficient centres were associated with positive elctrostatic potential. In all the compounds it was evident that the regions containing oxygen atoms displayed negative elctrostatic potentials. Hence, they were electron-rich centers, and were susceptible to electrophilic attacks and hydrogen bonding. Regions with postive elecrostatic potential were localized within the vicinity of the hydrogen atoms and the alkyl groups [42].

### 2.6. Pharmacokinetic and Drug-Likeness Studies

The ADMET profiling of the four top-ranked prenylated flavonoids were performed in order to elucidate their drug candidacy by considering important pharmacokinetic parameters, such as solubility, blood brain barrier permeability, human intestinal absorption, acute oral toxicity, and carcinoegenicity (Table 4).

The solubility value of a good drug candidate ranged between −6.5 and 0.5 [43]. All the top-ranked prenylated flavonoids had solubility values that ranged between these values, with arcommunol B showing the minimum solubility value at −4.7, while manuifolin H elicited the maximum solubility value at −3.392. Blood-brain barrier (BBB) was used to evaluate the potenials of drug lead molecules to enter into the central nervous system, while human intestinal absorption gave detailed information on the potentials of hit molecules to be absorbed into the blood stream through the intestinal section of the body. All the top ranked molecules possessed blood-brain barrier and human intestinal absorption properties. The values obtained for the acute oral toxicity and carcinogenicity suggested that the top-ranked glucokinase activators fulfilled the safety criteria for a drug candidate.

In terms of drug likeness properties, pan-assay interference compounds (PAINS), Linpiski’s, and Veber’s rules were considered. PAINS-like compounds were ligands that gave false positive bioactivity potentials during screening [44]. Lipinski’s rule of 5 (molecular weight ≤ 500, hydrogen bond acceptor ≤ 10, hydrogen bond donor ≤ 5, logP ≤ 5, and molar refractivity 40–130) and Veber’s rule (molecular weight 160–480, TPSA−140, molar refractivity 40–130, and the number of atoms 20–70) were important in identifying drug-like molecules. In our study, all the hit molecules passed the PAINS filter test, Lipinski’s, and Veber’s rule. Hence, they could be recommended as drug-like and possible therapeutic molecule as glucokinase activator.

## 3. Materials and Methods

### 3.1. Receptor and Ligand Preparation

The three-dimensional crystal structure of glucokinase with PDB ID 1V4S was downloaded from the protein data bank platform (http://www.rcsb.org/pdb/structure/1V4S, accessed on 20 October 2021). The protein was prepared for docking by removing all water molecules, co-crystallized ligand, co-factors, and ions, while charges and hydrogen were added to the protein using the built-in open babel plug-in tool in PyRx 0.8.

A total of 221 naturally occurring prenylated flavonoids (see Table S1) isolated from medicinal plants were downloaded in SDF format from the Pubchem database (https://pubchem.ncbi.nlm.nih.gov/, accessed on 20 October 2021). The energy minimization of the ligands was carried out under MMFF94x force field in the Open Babel plug-in tool of PyRx 0.8 tool and saved in PDB format for docking purposes.

### 3.2. Validation of Docking Protocol and Molecular Docking Studies

Prior to the molecular docking studies, a suitable docking protocol was developed by focusing on the enzyme’s active site and considering amino acid residues within 5 Å (Table 5). All water molecules, co-crystallized ligand, and co-factors were removed from the glucokinase enzyme. The co-crystallized ligand was re-docked at the enzyme’s binding pocket and its best conformational pose was selected, based on the RMSD value calculated. Additionally, the amino acid residues interacting with the re-docked ligand within 5 Å were examined with the PyMol tool.

Virtual screening was carried out by loading and converting each ligand and the glucokinase receptor from PDB to PDBQT using the Autodock plug-in functionality in the PyRx software. Polar hydrogens were added to the enzyme and non-polar hydrogen atoms were merged. Thereafter, the grid box size and dimension for the glucokinase enzyme were set at x = 38.8820, y = 14.8395, and z = 61.4901; x = 22.0193, y = 22.1167, and z = 23.2341, respectively. Docking was performed with the Autodock Vina plug-in in PyRx (GUI version 0.8) [45]. Results with the best binding energy and lowest RMSD value were chosen for the ligands. Thereafter, the hydrogen bond, hydrophobic, and pi-interactions protein-ligand complex of the top-ranked molecules were analysed with Discovery Studio Visualizer 2020.

### 3.3. Molecular Dynamics Simulation

Coordinates of the complexes with the top four ligands having the lowest binding free energy obtained from the docking results were selected to perform molecular dynamics simulations (MDS) using Desmond simulation package of Schrödinger software [46,47]. These complexes provided to Desmond were previously prepared using a protein preparation wizard. MDS was carried out to understand the binding stability and conformational changes of these complexes. In all runs, the NPT ensemble with 300 K temperature and a pressure 1.01325 bar was applied. The simulation length of 100 ns was carried out for the protein-ligand complexes and the snapshots were saved at an interval of 100 ps, resulting in a total of 400 snapshots for each of the four ligands.

During the simulations, the OPLS4 force field parameters were used and a cut-off radius of 9.0 Å was applied for analyzed short-range van der Waals and Coulomb interactions [48]. Nose-Hoover and Martyna-Tobias-Klein chain coupling scheme was used for the thermostat and barostat approaches, respectively [47]. The Particle-Mesh Ewald method [49] was used to evaluate long-range electrostatic interactions. The simple point-charge model [50] was used to explicitly describe the water molecules. The simulation interaction diagram tool incorporated in the Desmond MD package was used to analyse simulations of the root mean square deviations (RMSD), root mean square fluctuations (RMSF), and protein-ligand contact formed throughout the simulation period.

### 3.4. Theoretical Modelling and Optimization Studies

DFT analysis of the four compounds was carried out using the Spartan 14 programme containing functional B3LYP (Lee-Yang-Parr exchange-correlation functional method). Additionally, a 6–31G basis set was chosen for the DFT study [51]. During the calculations, the values of the frontier orbital energies were computed from the most established conformation of the compounds (See supplementary information S2).

### 3.5. Pharmacokinetic and Drug-Likeness Study

The ADMET properties of the hit molecules were estimated by loading their canonical smiles into admetSAR 2 online server (http://lmmd.ecust.edu.cn/admetsar2/, accessed on 20 October 2021) [52]. ADMET descriptors determined for each ligand if they included blood-brain barrier penetration, human intestinal absorption, solubility, carcinogenicity, and acute oral toxicity. These properties contributed tremendously to the drug candidacy and performance of phytochemicals in the treatment of diseases.

Furthermore, the drug-likeness potential of the hit molecules were evaluated by assessing their compliance with Linpiski’s and Veber’s rules [53,54].

## 4. Conclusions

In the present study, the roles of naturally occurring prenylated flavonoids as glucokinase activators were elucidated using computational methods. The molecular docking studies revealed arcommunol B, kuwanon S, manuifolin H, and kuwanon F as the top-ranked molecules and potential glucokinase activators. Molecular dynamics simulation and MM/GBSA calculations showed that good binding affinity existed between the active site of the glucokinase enzyme and the top-ranked prenylated flavonoids. The quantum chemical calculations revealed the promising pharmacological potentials of the top-ranked prenylated flavonoids as glucokinase activators. ADMET studies revealed arcommunol B, kuwanon S, manuifolin H, and kuwanon F as promising drug candidates for activating the glucokinase enzyme towards blood glucose regulation. The study further justified the usage of *Artocarpus communis* and *Morus alba* in the management of diabetes, and suggested glucokinase activation as one of the possible mechanisms of action in the management of diabetes by these plants. Further studies using in vitro and in vivo methods are recommended to establish the ligands as potent glucokinase activators.

## Figures and Tables

**Table 1:** Interaction analysis of arcommunol B, kuwanon S, manuifolin H and kuwanon F with the glucokinase enzyme.

Ligand Pubchem ID	Chemical Structure	Binding Energy (kcal/mol)	Hydrogen Bonding Interaction		Hydrophobic Interaction	Pi-Interaction
			Amino Acid Residue	Distance (Å)		
Arcommunol B 101781179	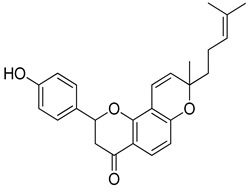	−10.1	Tyr61	2.19	Val62, Arg63, Pro66, Ile159, Ile211, Tyr214, Val452, Val455,	Val62, Arg63, Pro66, Ile159, Ile211, Tyr214, Met235, Val452, Val455
Kuwanon S 6450924	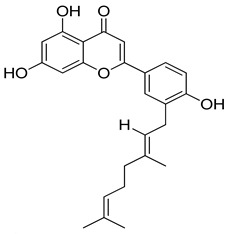	−9.6	-	-	Val62, Arg63, Pro66, Ile211, Tyr214, Val452, Val455, Ala456, Lys458, Lys459,	Val62, Arg63, Pro66, Ile211, Tyr214, Val452, Val455, Ala456, Lys458, Lys459.
Manuifolin H 15837463	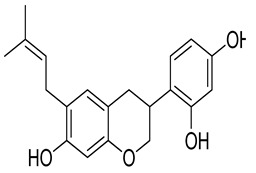	−9.5	Leu451	2.05	Val62, Arg63, Pro66, Ile211, Tyr214, Met235, Val452, Val455,	Val62, Arg63, Pro66, Ile211, Tyr214, Met235, Val452, Val455.
Kuwanon F 156149	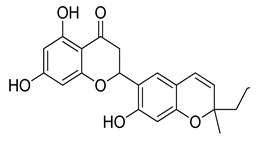	−9.4	Val452	2.82	Val62, Arg63, Pro66, Ile159, Ile211, Tyr214, Val452, Val455,	Val62, Arg63, Pro66, Ile211, Tyr214, Val452, Val455

**Table 2:** MMGBSA ΔG binding scores of the top-ranked molecules.

Ligand	MMGBSA ΔG Bind	MMGBSA ΔG Bind Coulomb	MMGBSA ΔG Bind Covalent	MMGBSA ΔG Bind Solvation Energy	MMGBSA ΔG Bind vdW
Arcommunol B	−70.23	−5.27	1.63	21.11	−57.91
Kuwanon S	−54.86	−9.12	2.23	17.20	−50.88
Manuifolin H	−37.34	−7.26	1.35	15.94	−29.89
Kuwanon F	−26.76	−8.64	0.97	14.48	−27.12

**Table 3:** Some calculated quantum chemical parameters for the compounds.

Ligands	E_HOMO_	E_LUMO_	ΔE	η (Chemical Hardness)	μ (Chemical Potential)	ω (Electrophilcity Index)
Arcommunol B	−5.70	−1.30	4.40	2.20	−3.5	2.78
Kuwanon S	−5.83	−1.68	4.15	2.08	−3.76	3.40
Manuifolin H	−5.19	−0.01	5.18	2.59	−2.60	1.31
Kuwanon F	−5.43	−1.31	4.12	2.06	−3.37	2.76

**Table 4:** Pharmacokinetics and drug-likeness studies of arcommunol B, kuwanon S, manuifolin H, and kuwanon F.

Ligands	Lipinski Rule Violation	Veber Rule Violation	PAINS Test	Solubility	BBB	HIA	Acute Oral Toxicity	Carcinogenicity
Arcommunol B	0	0	0	−4.700	+	+	2.347	-
Kuwanon F	0	0	0	−4.078	+	+	2.612	-
Kuwanon S	0	0	0	−4.351	+	+	2.208	-
Manuifolin H	0	0	0	−3.392	+	+	1.8	-

**Table 5:** Enzyme and the amino acid residues at its binding site.

PDB ID	Residues within 5 Å	Native Ligand
1V4S	Tyr61, Val62, Arg63, Ser64, Thr65, Pro66, Gln98, Ile159, Met210, Ile211, Tyr214, Tyr215, His218, Cys220, Glu221, Met235, Arg250, Leu451, Val452, Val455, Ala456	2-Amino−4-fluoro−5-[(1-methyl−1H-imidazol−2-yl)sulfanyl]-N-(1,3-thiazol−2-yl)benzamide

## Data Availability

Data is available in this article and Supplementary Materials.

## Supplementary Materials

The following are available online. Table S1: List of compounds and their PubChem IDs; File S1: Formulas used for quantum chemical calculations.

## Sample Availability

Samples of the compounds are not available from the authors.
