# Supplementary material for: Elucidating the Glucokinase Activating Potentials of Naturally Occurring Prenylated Flavonoids: An Explicit Computational Approach
Source: Molecules. 2021 Nov 28;26(23):7211. doi: 10.3390/molecules26237211 (PMC8659159; doi:10.3390/molecules26237211)
Supplement: Supplementary file 1 [file molecules-26-07211-s001.zip › molecules-1449993-supplementary.pdf]

Elucidating the glucokinase activating potentials of naturally occurring prenylated flavonoids: an explicit computational approach

Table S1: Prenylated flavonoids and their PubChem IDs

S/N	Compounds	Pubchem ID
1	1-methoxyerythrabyssin II	24761044
2	2'-Methoxykurarinone	11982641
3	3′-O-methyldiplacone	14539951
4	5-Hydroxysophoranone	42607927
5	6-prenylnaringenin	155094
6	8-lavandulylkaempferol	16083184
7	8-prenylquercetin	9799499
8	Abyssinin II	442457
9	Abyssinone IV	4063835
10	Alopecurone G	42607826
11	Alpinumisoflavone	5490139
12	Artelastin	399488
13	Artelastochromene	5471310
14	Artobiloxanthone	46887866
15	Artocarpesin	399491
16	Artocarpin	5458461
17	Artoindonesianin_B	10096171
18	Artoindonesianin P	10316935
19	Artoindonesianin S	5320444
20	Artonin E	5481962
21	Artonin J	44258663
22	Artonin K	15340661
23	Artonin L	44258662
24	Artonin P	44258658
25	Artonin S	44258666

26	Artonin T	44258664
27	Artonin U	44258358
28	Artonin V	129687399
29	Artonin Y	15541482
30	Bicolosin A	56668791
31	Bicolosin B	56665359
32	Bitucarpin_A	21576494
33	Bitucarpin B	101756926
34	Bolusanthol B	10594416
35	Bolusanthol_C	12972420
36	Caeruleanone_A	102231361
37	Caeruleanone B	102231362
38	Caeruleanone C	102231363
39	Chaplashin	90472917
40	Crotafuran A	10245439
41	Crotafuran E	639695
42	Cudraflavone B	5319925
43	Cudraflavone C	5319924
44	Cycloartobiloxanthone	10342859
45	Cycloartocarpin A	44258302
46	Cyclocommunin	9979523
47	Cyclocommunol	10315987
48	Deoxymiroestrol	9927999
49	Dereticulatin	42607939
50	Derrisin	10274158
51	Desmodianone_D	11102162
52	Discoloranone_A	11995376
53	Erybreadin C	21147013
54	Erybreadin D	46880036
55	Erythrabyssin II	5086400
56	Erycristagallin	10362969
57	Euchrestaflavanone A	484588

58	Erylatissin A	11739635
59	Erylatissin_B	11186717
60	Erylatissin_C	11382659
61	Erylatissin G	11186717
62	Erysenegalensein_N	49768588
63	Erysenegalensein_O	49768587
64	Erystagallin A	10410005
65	Erysubin E	637080
66	Erysubin F	12051847
67	Erythribyssin O	46861837
68	Erythrinin C	44257283
69	Eryvarin B	101010400
70	Eryvarin_D	15546808
71	Eryvarin_F	637295
72	Eryvarin N	102208346
73	Eryvarin O	102208347
74	Eryzerin_B	10862753
75	Eryzerin_C	10092034
76	Eryzerin D	11761017
77	Euchrenone_b10	402594
78	Flavenochromane A	11351168
79	Fremontin	5487268
80	Hildecarpin	442773
81	Furowanin_A	11582970
82	Glabrisoflavone	5378945
83	Glabrol	11596309
84	Griffonianone B	12972368
85	Griffonianone_D	11004658
86	Griffonianone_F	101385741
87	Indicanine A	54688677
88	Indicanine B	54712408
89	Indicanine C	10736576

90	Indicanine E	636496
91	Isocyclomulberrin	5316260
92	Isokurarinone	5318581
93	Isosophoranone	10478290
94	Isoxanthohumol	513197
95	Khonklonginol B	44179864
96	Khonklonginol C	44179860
97	Khonklonginol D	44179865
98	Khonklonginol E	44179861
99	Khonklonginol G	44178660
100	Khonklonginol H	44179862
101	Kraussianone 3	102510321
102	Kuraridine	9954815
103	Kuraridinol	5318880
104	Kurarinone	11982640
105	Kushenol A	44563121
106	Kushenol B	5318891
107	Kushenol C	5481237
108	Kushenol D	5318893
109	Kushenol E	127234
110	Kushenol G	44259516
111	Kushenol I	20832634
112	Kushenol K	5318897
113	Kushenol L	21721878
114	Kushenol N	381851
115	Kushenol P	10742453
116	Kushenol R	10835998
117	Kushenol T	10598514
118	Kushenol U	11796501
119	Kushenol V	10572194
120	Kushenol W	42608033
121	Kushenol X	10599228

122	Kuwanon C	5481958
123	Kuwanon A	44258296
124	Kuwanon B	44258295
125	Kuwanon F	156149
126	Kuwanon S	6450924
127	Kuwanon T	15231527
128	Kuwanon_U	46209782
129	Laburnetin	15237156
130	Leachianone A	44593449
131	Leachianone G	5275227
132	Lespecyrtin E3	25208431
133	Lespecyrtin E5	25208432
134	Lespeflorin G5	25227611
135	Lespeflorin G10	25242998
136	Lupiwighteone	5317480
137	Manuifolin F	10596805
138	Manuifolin H	10596805
139	Millewanin E	11407932
140	Millewanin G	11662094
141	Millewanin H	11597321
142	Lupalbigenin	10001388
143	Lupinifolin	10250777
144	Morusin	5281671
145	Norkurarinol A	44563159
146	Nymphaenol A	639465
147	Obovatin	13940733
148	Papyriflavonol A	10343070
149	Phaseollidin	119268
150	Puemiricarpene	10089353
151	Pomiferin	4871
152	Pumilaisoflavone A	14035924
153	Pumilaisoflavone B	14035925

154	Rautandiol A	11674519
155	Rotenone	6758
156	Sanggenon K	44559964
157	Sanggenon N	42608044
158	Secundifloran	10091530
159	Senegalensin	124035
160	Sigmoidin A	73204
161	Sophoflavescenol	9929189
162	Sophoraflavanone A	6475921
163	Sophoflavanone K	16083183
164	Sophoraflavanone G	72936
165	Sophoranone	441767
166	Sophorapterocarpan A	14017299
167	Tetrapterol F	100935759
168	Tetrapterol H	100935760
169	Tetrapterol I	100935761
170	Tomentodiplacone	24861959
171	Tomentodiplacone B	24861960
172	Tomentodiplacone	24861959
173	Triquetrumone A	102216287
174	Kuwanon E	6440408
175	Propolin A	10411087
176	Schizolaenone C	46871853
177	6-Geranylchrysin	5281943
178	Alpinumisoflavone	5490139
179	Arcommunol B	101781179
180	Artoheterophyllin H	102133563
181	Atalantoflavone	14162621
182	Bavachin	14236566
183	Broussoflavonol A	21676157
184	Broussoflavonol B	480828
185	Candenatenin F	44254971

186	Cannflavin A	10071695
187	Carpachromene	10449654
188	Cyclomulberrin	11742872
189	Dalvelutinane A	132497058
190	Dalvelutinane B	132497059
191	Discoloranone	11995377
192	Glyasperin F	392442
193	Heminitidulan	629585
194	Hiravanone	14542255
195	Honyucitrin	44257865
196	Icaritin	5318980
197	Isodiscoloranone A	11995289
198	Khonklonginol A	44179863
199	Leiocin	630752
200	Leiocinol	596540
201	Licoagroisoflavone	636883
202	Lonchocarpusone	44559034
203	Maackiapentone	25231268
204	Macaranone B	42642846
205	Nitidulin	630902
206	Neobavaisoflavone	5320053
207	Podoverine A	13911680
208	Sanggenon A	156707
209	Schizolaenone C	46871853
210	Sanggenon M	44559964
211	Sinoflavonoid A	77106371
212	Sinoflavonoid B	77106372
213	Ulexone A	14583600
214	Vogelin C	44448192
215	Vogelin D	636894
216	Vogelin E	10904337
217	Vogelin F	11143123

218	Vogelin G	11026226
219	Vogelin H	101396887
220	Vogelin I	101396888
221	Yukovanol	14542257

Supplementary File S1

Formulas used for quantum chemical calculations.

$$\Delta E = E_{LUMO} - E_{HOMO} \quad (1)$$

$$\eta = \frac{1}{2}(E_{LUMO} - E_{HOMO}) \quad (2)$$

$$\mu = \frac{1}{2}(E_{HOMO} + E_{LUMO}) \quad (3)$$

$$\omega = \frac{\mu^2}{2\eta} \quad (4)$$
